# Domestic

**Published:** 1841-04-10

**Authors:** 


### DOMESTIC.

*Jefferson Medical College.*—The Trustees of this Institution, on Tuesday last, re-organized the Medical Faculty by electing the following gentlemen Professors:

Robley Dunglison, M. D.—Institutes of Medicine and Medical Jurisprudence.

Robert M. Huston, M. D.—Materia Medica and General Therapeutics.

Joseph Pancoast, M. D.—Anatomy.

John K. Mitchell, M. D.—Practice of Medicine.

Charles D. Meigs, M. D.—Obstetrics and Diseases of Women and Children.

Franklin Bache, M. D.—Chemistry.

Jacob Randolph, M. D.—Practice of Surgery.

Thomas D. Mütter, M. D.—Institutes of Surgery.

---

*Death of Professor Colhoun.*—Samuel Colhoun, M. D., Professor of Materia Medica in the Pennsylvania Medical College, died on Wednesday morning, April 7th,—a learned and estimable physician.

---

*The Medical Schools.*—In the University of Pennsylvania, during the past session, there were 410 students; at the recent commencement, 166 graduates; in the Transylvania University, at Lexington, Ky., 254 students, 62 graduates; in the Medical Institute of Louisville, Ky., 205 students; in the Jefferson Medical College, 163 students, 57 graduates; in the Pennsylvania Medical College, 112 students, 43 graduates; in the Geneva Medical College, 136 students, 34 graduates; in the Albany Medical College, 122 students, 29 graduates; in the Harvard University at Boston, 88 students.

*The Chair of Theory and Practice of Medicine in the Transylvania University.*—The chair of the theory and practice of medicine in the medical department of this institution is at present vacant, and with a view to fill it in the best possible manner, the board of trustees invite applications for the appointment from the members of the medical profession.

The communications on the subject must be addressed to the *Dean of the Medical Faculty of Transylvania University*, and come to hand before the first of June next, when the appointment will be made. The name of no one but the successful candidate will be made public.

### HEALTH OF THE CITY.

INTERMENTS *in the City and Liberties of Philadelphia, from the 27th of March to the 3d of April*, 1841.

| Diseases. | Adults. | Children. |
|---|---|---|
| Abscess of brain, | 0 | 1 |
| —— lumbar, | 1 | 0 |
| Apoplexy, | 1 | 0 |
| Cancer, | 1 | 0 |
| —— breast, | 1 | 0 |
| Croup, | 0 | 3 |
| Consumption of the lungs, | 15 | 4 |
| Contusion on the abdomen, | 1 | 0 |
| Convulsions, | 0 | 4 |
| Cyanosis, | 0 | 1 |
| Diarrhœa, | 1 | 0 |
| Dropsy, | 1 | 0 |
| —— head, | 0 | 4 |
| —— breast, | 0 | 1 |
| Disease of heart, | 1 | 0 |
| —— lungs, | 1 | 0 |
| —— liver, | 1 | 0 |
| Drowned, | 0 | 1 |
| Debility, | 1 | 2 |
| Enlargement of the heart, | 0 | 1 |
| Fever, remittent, | 2 | 0 |
| —— typhus, | 1 | 0 |
| —— puerperal, | 1 | 0 |
| —— scarlet, | 0 | 1 |
| Gangrene of lungs, | 1 | 0 |
| Gangrenous sore throat, | 0 | 1 |
| Hæmorrhage, | 1 | 0 |
| Inflammation of the brain, | 0 | 4 |
| —— bronchi, | 1 | 1 |
| —— lungs, | 6 | 6 |
| —— stomach, | 1 | 0 |
| —— bowels, | 0 | 2 |
| | — | — |
| *Carried forward*, | 40 | 36 |
| *Brought forward*, | 40 | 36 |
| Inflammation of breast, | 0 | 1 |
| —— bladder, | 1 | 0 |
| —— peritonæum, | 2 | 0 |
| Marasmus, | 0 | 2 |
| Measles, | 0 | 2 |
| Old age, | 1 | 0 |
| Palsy, | 2 | 0 |
| Pleurisy, | 2 | 0 |
| Rheumatism, | 1 | 0 |
| Small pox, | 2 | 2 |
| Spina bifida, | 0 | 1 |
| Still-born, | 0 | 8 |
| Syphilitic cachexia, | 1 | 0 |
| Unknown, | 1 | 1 |
| Violence, | 0 | 1 |
| | — | — |
| Total, 107— | 53 | 54 |

Of the above, there

| | |
|---|---|
| were under 1 year | 29 |
| From 1 to 2 | 7 |
| 2 to 5 | 10 |
| 5 to 10 | 6 |
| 10 to 15 | 1 |
| 15 to 20 | 1 |
| 20 to 30 | 8 |
| 30 to 40 | 20 |
| 40 to 50 | 12 |
| 50 to 60 | 3 |
| 60 to 70 | 5 |
| 70 to 80 | 2 |
| 80 to 90 | 2 |
| 90 to 100 | 1 |
| Total, | 107 |

In the above are included 6 people of colour, and 3 interments from the alms-house.

*Weekly Report of Interments in the City and County of New York, from the 27th of March to the 3d of April*, 1841.—Aneurism 1; Abscess 1; Bleeding from lungs 2; Burned or scalded 1; Cancer 2; Consumption 31; Convulsions 12; Croup or Hives 3; Delirium tremens 3; Dropsy in the head 8; Drowned 1; Erysipelas 2; Fever 2; do. scarlet 7; do. typhoid 2; do. puerperal 3; do. intermittent 1; indigestion 6; intemperance 1; inflammation 1; do. of throat 3; do. of brain 5; do. of liver 4; do. of chest 2; do. of lungs 18; do. of bowels 7; do. of stomach 1; Marasmus 6; Measles 6; Old age 4; Small Pox 3; Scrofula 1; Teething 1; Unknown 3.

Ages—Of 1 year and under, 29; between 1 and 2, 17; 2 and 5, 24; 5 and 10, 9; 10 and 20, 11; 20 and 30, 19; 30 and 40, 17; 40 and 50, 7; 50 and 60, 10; 60 and 70, 4; 70 and 80, 4; 80 and 90, 3.

34 men—34 women—43 boys—43 girls. Total, 154.

